# Managing Infections in Burn Patients: Strategies and Considerations for Antimicrobial Dosing

**DOI:** 10.3390/ebj6040053

**Published:** 2025-10-01

**Authors:** Abdullah F. Alharthi, Khalid Al Sulaiman, Sultan Alotaibi, Rahaf Alqahtani, Nader Damfu, Aisha Alharbi, Sufyan Alomair, Haifa A. Alhaidal, Ohoud Aljuhani

**Affiliations:** 1 Department of Clinical Pharmacy, College of Pharmacy, Shaqra University, Al-Dawadmi Campus, Al-Dawadmi 11961, Saudi Arabia; 2 Pharmaceutical Care Services, King Abdulaziz Medical, Riyadh 11481, Saudi Arabia; 3 College of Pharmacy, King Saud bin Abdulaziz University for Health Sciences, Riyadh 14611, Saudi Arabia; 4 Clinical Trial Management, King Abdullah International Medical Research Center, Riyadh 14611, Saudi Arabia; 5 Saudi Critical Care Pharmacy Research (SCAPE) Platform, Riyadh 11481, Saudi Arabia; 6 Saudi Society for Multidisciplinary Research Development and Education (SCAPE Society), Riyadh 11481, Saudi Arabia; 7 Pharmaceutical Care Services, King Abdulaziz Medical City, Ministry of the National Guard-Health Affairs, Jeddah 22384, Saudi Arabia; 8 King Abdullah International Medical Research Center, Jeddah 22384, Saudi Arabia; 9 King Saud bin Abdulaziz University for Health Sciences, Jeddah 22384, Saudi Arabia; 10 Infection Prevention and Control Department, King Abdulaziz Medical City, Ministry of the National Guard-Health Affairs, Jeddah 22384, Saudi Arabia; 11 Department of Pharmacy Practice, College of Clinical Pharmacy, King Faisal University, Al-Ahsa 36362, Saudi Arabia; 12 Pharmaceutical Services Administration, King Saud Medical City, Riyadh 12746, Saudi Arabia; haifaalhaidal@gmail.com; 13 Department of Pharmacy Practice, Faculty of Pharmacy, King Abdulaziz University, Jeddah 22252, Saudi Arabia

**Keywords:** burn, infections, pharmacokinetics, pharmacodynamics, antimicrobial dosing

## Abstract

Burn injuries are a major cause of morbidity and mortality, largely due to complications such as infection. Impairment of the immune system following burns increases susceptibility to both internal and external infections, underscoring the need for effective infection control strategies in burn care. In addition, burn patients frequently exhibit profound alterations in drug pharmacokinetics and pharmacodynamics (PK/PD), particularly during the resuscitation and hypermetabolic phases. In the resuscitation phase, increased capillary permeability and reduced cardiac output can prolong drug distribution, delay therapeutic response, lower peak plasma concentrations, and slow elimination. In contrast, the hypermetabolic phase is characterized by elevated catecholamine levels and enhanced tissue perfusion, which accelerate drug distribution and clearance. These physiological changes often necessitate antimicrobial dose adjustments to maintain therapeutic efficacy. This review emphasizes the critical importance of infection prevention and management in burn patients, with a focus on optimizing antimicrobial dosing and therapeutic monitoring in the context of PK/PD alterations.

## 1. Background

Burn injuries are considered a challenging crisis for both patients and healthcare personnel due to the multitude of problems that can occur during and after the treatment procedure. The skin acts as a vital defense against external microorganisms; however, when burns occur, the skin’s protective function is compromised, allowing microorganisms to enter the affected area and proliferate within the damaged tissue. Furthermore, burns can compromise the immune system, thereby increasing vulnerability to internal and external infections, a significant cause of morbidity and mortality in this population. The onset or progression of infection in these patients significantly influences mortality, highlighting the critical need for effective infection control strategies in burn care [1].

Moreover, the bacterial flora found within burn wounds is a dynamic entity that undergoes significant changes over time. Initially, these wounds are considered sterile, but within a few days, Gram-positive cocci such as methicillin-resistant *Staphylococcus aureus* (MRSA) and *Streptococcus pyogenes*, start to colonize the wounds from deeper structures such as hair follicles and glands. In the second phase, a Gram-negative shift occurs, where *Pseudomonas aeruginosa*, *Escherichia coli*, and *Proteus* are the predominant isolates. The emergence of multidrug-resistant (MDR) bacteria has posed a significant challenge in managing burns [2,3]. A recent study on severely burned patients revealed that the most frequently isolated bacteria were *Pseudomonas aeruginosa*, followed by *Staphylococcus aureus*, *Klebsiella* spp., and *Acinetobacter baumannii* [4].

Furthermore, burn patients are vulnerable to multiple pharmacokinetic and pharmacodynamic (PK/PD) changes, particularly in response to shock and the hypermetabolic state characteristic of burn injury. Generally, burn injuries can be divided into two stages based on physiological changes [5]. In the initial resuscitative phase, occurring within the first 48 h post-injury, pharmacokinetic alterations—such as prolonged distribution, delayed onset of action, reduced maximum serum concentration (C_max_), and slower drug elimination—are expected due to factors like capillary hyperpermeability and decreased cardiac output [6]. Subsequently, during the hypermetabolic stage—characterized by elevated catecholamines and increased tissue perfusion, pharmacokinetic changes such as faster distribution and shorter elimination half-life (t_1/2_) might occur, necessitating antimicrobial dosing adjustments to maintain therapeutic efficacy [7,8].

As a result of these unique features of PK/PD alterations, optimizing antimicrobial dosing and selection is considered crucial to enhance the outcome and prevent the risk of microbial resistance in this population. Despite the recognized significance of infection prevention and management in patients with burn injury, the evidence base on this topic is still developing, particularly considering the challenges posed by the significant changes in PK/PD of antimicrobials and the increased risk of infections, including acquiring multidrug-resistant organisms (MDR), which could further limit their therapeutic options. Therefore, this review aims to underscore the critical importance of infection prevention and management in burn patients, emphasizing the need for appropriate antimicrobial dosing and monitoring tailored to the unique PK/PD profile of burn patients. Table 1 summarizes studies on antimicrobial agents in burn patients.

## 2. Method

Articles published via PubMed and Scopus from inception till January 2025 were evaluated for inclusion using the following terms: “Burn injuries”, “Infection control”, “Pharmacokinetics”, “Pharmacodynamics”, and “Antimicrobial dosing”. Additional references, including abstracts and conference posters, were identified through a manual search employing the same search terms. Only articles published in English that focused on adult burn patients and directly addressed infection prevention, antimicrobial management, or pharmacological considerations were included in this review. Original research, including randomized controlled trials (RCTs), observational studies (including retrospective, prospective, and case series/case reports), and pharmacokinetic studies, was included. Selected in vitro and in vivo studies were included when they considered the only available evidence on certain aspects. We excluded non-original research (e.g., review articles) and studies that primarily discussed non-pharmacological interventions, psychological aspects, or caregiver experiences related to burn victims. The included studies were evaluated using the levels of evidence provided by the Oxford Centre for Evidence-Based Medicine (OCEBM)’s.

## 3. Infection Prevention and Control in Burn Patients

Wounds caused by severe burns are susceptible to bacterial colonization. The use of systemic prophylactic antibiotics for severe burn patients is still controversial. The majority of the studies did not result in a significant reduction in wound infections by using systemic prophylactic antibiotics. Therefore, it is not recommended to use them for this purpose [35,36,37].

In contrast, one study showed that using prophylactic first-generation cephalosporins or ampicillin/sulbactam may decrease 28-day in-hospital mortality in mechanically ventilated patients with severe burns. However, the unknown impact of this study on antimicrobial resistance, combined with its retrospective design, would limit its applicability to practice [9]. Another study (which included 40 patients) showed that trimethoprim-sulfamethoxazole prophylaxis reduced the incidence of MRSA pneumonia in burn patients, but the study was limited by its small sample size and uncertain risk of bias [10].

As for prophylactic topical antibiotics, such as silver sulfadiazine, there is no evidence to support the use of these agents in significantly reducing the risk of burn wound infection compared to other topical preparations, dressings, placebos, or no treatment [35].

Patients with burns are at high risk of acquiring infections. Therefore, sustained efforts to maintain infection control and attention to preventing the transmission of pathogenic microorganisms are highly recommended. Moreover, maintaining a clean hospital environment through proper hand hygiene, microbial monitoring, regular cleaning, effective air filtration, and adequate ventilation would help reduce infections in burn patients [37].

## 4. Infection Treatment in Burn Patients

### 4.1. Treatment for Bacterial Infection: General Considerations

**Figure 1:**
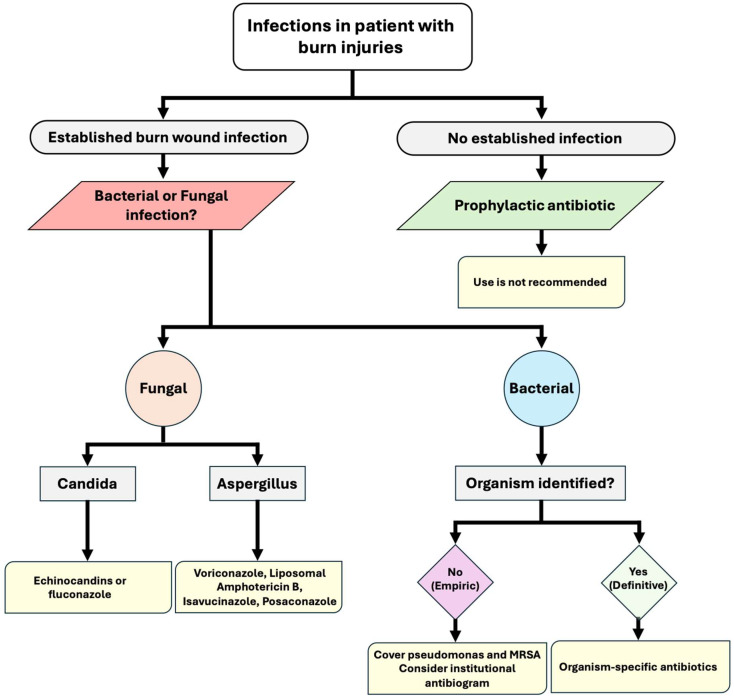
Algorithm for infection management in patients with burn injuries.

In managing patients with severe burn wound infection, the immediate priorities involve restoring hemodynamic stability. Simultaneously, broad-spectrum antibiotics are initiated based on the institution’s antibiogram [38], with a focus on antipseudomonal agents such as piperacillin/tazobactam or fourth-generation cephalosporins [39,40,41]. Burn units experience a high prevalence of methicillin-resistant *Staphylococcus aureus* (MRSA), with infection rates exceeding 50%. Considering this concerning epidemiology, empiric antibiotic therapy should prioritize vancomycin in alignment with the local antibiogram (Figure 1) [42]. Whenever possible, broad-spectrum therapy should be transitioned to organism-specific antibiotics to mitigate the risk of superinfections with resistant organisms (Table 2) [38,39].

The patient’s clinical response dictates the duration of antibiotic therapy and should be tailored upon definitive diagnosis through burn wound cultures and histopathology [38]. Once the patient is stabilized, thorough surgical debridement is crucial, aiming to remove all infected tissue to a healthy base, confirmed by intraoperative biopsy. Reassessment and repeat debridement within 24–48 h are also necessary to ensure complete removal of necrotic tissue. Finally, definitive wound closure should be achieved with autologous skin grafts whenever feasible, as early excision and autografting are associated with better graft take, lower infection rates, shorter hospitalization, and improved survival [43].

While systemic antibiotics may reach some viable tissues, their penetration into necrotic areas is limited, potentially leading to the selection of resistant bacterial strains among the diverse microbial flora colonizing the wound. Consequently, ongoing pharmacokinetic and pharmacodynamic evaluations by a qualified clinical pharmacist are crucial to optimize drug dosing regimens, ensuring both safety and efficacy [44,45].

### 4.2. Treatment for Fungal Infections: General Considerations

Invasive fungal infections in burn patients were associated with a 10-fold increase in 1-year mortality compared to burn patients without fungal infections (risk ratio 9.8, *p* < 0.0001), and an incremental increase in percent total body surface area (%TBSA) burns was strongly associated with fungal infection mortality [46]. Fungal colonization in intensive care patients with burns reached up to 16.1% [47]. Overall, Candida species (Albicans and non-Albicans) and Aspergillus can cause fungal infections in burn patients [3]. Candida species are reported to be the most common bloodstream infections, even more prevalent than bacterial infections, in burn patients [48].

The choice of antifungal treatment agent depends on the causative fungal infection. For Candida infections, echinocandins and fluconazole are commonly used, while voriconazole, isavuconazole, and posaconazole are preferred for managing invasive Aspergillus infections. Liposomal amphotericin B offers broad coverage and can be used to treat both Candida and Aspergillus infections [49,50]. The pharmacokinetics and pharmacodynamics of liposomal amphotericin B, fluconazole, posaconazole, and voriconazole are significantly altered in burn patients, necessitating higher adjusted dosing to treat fungal infections in these populations [51].

## 5. Appropriate Antimicrobials (Dosing, Pharmacokinetic Considerations, and Monitoring)

### 5.1. Antibiotics

#### 5.1.1. Cefepime

Many studies investigated how burn severity and patient-related characteristics can affect cefepime’s drug levels in burn patients. The normal cefepime dose for healthy people is 2 g every 12 h. However, some studies suggest that this may not be sufficient for burn patients, and suboptimal trough concentrations may occur [11]. To overcome this, more aggressive strategies such as shortened intervals or continuous infusions have been proposed [12,13]. Based on specific PK/PD targets and burn severity, various regimens have also been proposed [52]. One study has demonstrated the efficacy of therapeutic drug monitoring (TDM) in achieving targeted cefepime exposures [14]. However, more studies are needed to determine the appropriate dose of cefepime in burn patients.

#### 5.1.2. Ceftazidime

Numerous studies have investigated ceftazidime’s pharmacokinetic (PK) profile across various patient groups, suggesting the need for precise dosing strategies to achieve optimal drug exposure [15,16]. While early studies lacked specific dosing recommendations. Conil et al. later addressed this gap by demonstrating that traditional intermittent infusions frequently failed to achieve desired PK/PD targets, particularly in burn patients. Their findings confirmed the potential benefit of shortening dosing frequencies or continuous infusions, especially for bacteria with high minimum inhibitory concentration (MIC) [12,17]. Moreover, Le Floch et al. reported high target attainment with continuous infusion, albeit without providing specific details regarding implementation [53]. However, an old study demonstrated that intermittent infusions achieved favorable concentrations within tissues and burned blister fluids [54]. Collectively, these studies assure the significant role of adjusting ceftazidime regimens based on patient characteristics and PK/PD targets to achieve the optimal therapeutic regimen. Based on the limited evidence, an approach is to give a loading dose of ceftazidime followed by 1 g every 4 h as intermittent infusions, or to give ceftazidime as a 24 h continuous infusion of 6 g, for which the continuous infusion is typically preferred for maintaining therapeutic concentrations.

#### 5.1.3. Piperacillin/Tazobactam

Several PK/PD studies of piperacillin-tazobactam in burned patients have evaluated different dosing regimens and PK/PD targets, demonstrating significant variability in the PK parameters of piperacillin-tazobactam. One study suggests that 4.5 g every 6 h via intermittent infusion may be effective in burn patients [18]. However, others report that higher doses and prolonged infusion durations may be required for patients with augmented renal clearance [55,56]. Additionally, some studies report that the use of TDM increases the probability of target attainment (PTA), though further research is warranted [14,19,20].

#### 5.1.4. Meropenem

Multiple studies have investigated the pharmacokinetics of meropenem in burn injury. Doh et al. demonstrated insufficient meropenem efficacy with standard dosing in burn patients [21]. Subsequent studies supported the use of higher doses and continuous infusion for increased effectiveness. Ramon-Lopez et al. recommended a continuous infusion of 6 g over 24 h for pathogens with higher MICs. Similarly, Selig et al. found that continuous infusion improved efficacy with Augmented renal clearance (ARC) and higher MICs [57,58]. In contrast, studies by Messiano et al. and Corcione et al. observed favorable outcomes only against pathogens with low MIC [22,59]. More recently, a study showed that the PTA increased when the treatment was guided by TDM [20].

#### 5.1.5. Imipenem–Cilastatin

Different regimens and dosages have been utilized to examine the pharmacokinetics of imipenem–cilastatin in burn patients. Boucher et al. reported that PK parameters are comparable to those observed in healthy volunteers [23]. A daily dose of 2 g for patients with normal renal function and 1 g for patients with renal impairment achieved 100% PTA for bacteria with low MIC [60]. Moreover, pharmacokinetics studies of imipenem–cilastatin in burn patients receiving continuous renal replacement therapy (CRRT) have produced variable recommendations. One study recommended a high-dose regimen of 1 g every 6 h [23], while another study suggested a lower dose of 0.5 g every 6 h for low MIC [61]. The role of TDM has been studied and was found to be effective in guiding antibiotic treatment [20].

#### 5.1.6. Aztreonam

Two studies evaluated the PK of aztreonam in burn patients. The findings showed altered PK that could be due to an increased volume of distribution and clearance [24]. A dosing regimen of 2 g as a loading dose, followed by 8 g administered as a continuous infusion over 24 h, was suggested; however, specific PK/PD targets were not proposed [25].

#### 5.1.7. Novel Beta-Lactam in Combinations with Beta-Lactamase Inhibitor

A pharmacokinetic study of critically ill patients with multiple comorbidities and complications showed that a high dose of ceftazidime/avibactam may be necessary for patients with severely reduced kidney function [25]. In contrast, the use of ceftolozane-tazobactam, meropenem–vaborbactam, and imipenem–cilastatin–relebactam in burn patients has not been well studied yet.

#### 5.1.8. Ciprofloxacin

In burn patients with normal kidney function, the recommended dose of ciprofloxacin is 400 mg every 8 h, infused over 60 min [26]. The primary PK/PD parameter is area under the curve (AUC_0–24_): MIC, with favorable clinical outcomes reported with a target PK/PD of AUC_0–24_: MIC > 125:1 in critically ill patients [26]. Moreover, a pharmacokinetic study that evaluated a ciprofloxacin 400 mg intravenously (IV) every 8 h dose in burn patients showed that this regimen achieved attainment of an AUC/MIC ratio of 125 or more for infections caused by organisms with an MIC ≤ 0.25 µg/mL; however, the type of organism was not mentioned in the study [26].

#### 5.1.9. Levofloxacin

Levofloxacin is administered as 750 mg IV every 24 h over 90 min in burn patients’ infections [27]. Similarly to ciprofloxacin, levofloxacin’s PK/PD target is AUC_0–24_: MIC > 125:1 in critically ill patients [27]. This regimen achieved a probability of target attainment of greater than 90% against Gram-negative bacteria with MICs of 0.5 μg/mL or less and against Gram-positive bacteria with MICs of 1 μg/mL or less. Nevertheless, using this levofloxacin regimen in Gram-negative pathogens with MICs higher than 0.5 μg/mL was associated with inadequate achievement of the desired PK/PD target, and alternative antibiotic regimens are recommended in such cases [27].

#### 5.1.10. Vancomycin

In burn patients, a loading dose of vancomycin is typically 25 to 30 mg/kg, and a total daily dose of 40–70 mg/kg/day administered every 6–12 h is likely required to achieve a target serum trough level of 10 to 20 mg/L. A suggested vancomycin dosing of 20 mg/kg intravenously every 8 h is recommended to achieve this target level in those patients [62,63]. The desired vancomycin target PK/PD parameter is an AUC_0_–_24_:MIC ratio of greater than 400:1 in methicillin-resistant *Staphylococcus aureus* infections with a vancomycin MIC of 1 mg/L [62]. However, studies on other microorganisms are still scarce. Moreover, vancomycin would require frequent monitoring of serum creatinine and obtaining of vancomycin levels in burn patients [63].

#### 5.1.11. Daptomycin

A dose of 10–12 mg/kg of daptomycin is recommended intravenously every 24 h in burn patients [28,64]. Another approach in this population is a fixed daptomycin dose of 750 mg IV every 24 h [64]. In a pharmacokinetic study of daptomycin in burn patients, it was reported that a daptomycin dose of 6 mg/kg IV daily for *Staphylococcus aureus* infection resulted in a 44% and 39% decrease in AUC and Cmax, respectively [29]. Therefore, a suggested daptomycin dose of 10–12 mg/kg intravenously every 24 h is recommended and likely needed to achieve the target daptomycin PK/PD target of AUC_0–24_: MIC > 600:1 [28,64]. As daptomycin is known to cause rhabdomyolysis, baseline and weekly monitoring of creatine kinase (CK) levels is recommended [28].

#### 5.1.12. Linezolid

A linezolid dose of 600 mg IV every 12 h is still recommended; however, safety and efficacy studies of high-dose linezolid in burn patients are still lacking [30]. Severe burn injury has been associated with a 50% reduction in linezolid AUC_0_–_24_ compared with non-burn patients [30]. A pharmacokinetic study evaluating a regimen of 600 mg IV every 8 h demonstrated an 87.5% increase in time above the minimum inhibitory concentration (T > MIC); however, due to considerable variability in drug concentrations among those patients, TDM is advised to minimize the risk of treatment failure or toxicity. In the absence of TDM, this high dose is not recommended [65].

#### 5.1.13. Colistin

The recommended dose of colistin in burn patients is 150 mg of Colistin Base Activity (CBA) every 12 h, infused over 30 min [31]. One pharmacokinetic study that included 50 burn patients showed that the t_1/2_ of colistin (6.6 h vs. 5 h) and volume of distribution (81.1 L vs. 67.9 L) were increased compared to healthy non-burn patients. In the same study, colistin was administered as 150 mg of CBA every 12 h [62]. However, administration of higher doses might put the patients at risk of nephrotoxicity, and further studies are needed [31].

#### 5.1.14. Aminoglycosides

Multiple studies have evaluated the pharmacokinetics of aminoglycosides (amikacin, gentamicin, and tobramycin), various dosing regimens, and therapeutic drug monitoring in burn patients. Aminoglycosides have a high-volume distribution in burn patients, requiring higher doses to achieve target serum peak concentrations [32]. For once-daily dosing of aminoglycosides, amikacin 20 mg/kg administered intravenously once daily was likely to achieve the target peak concentration (Cmax) [33].

Regarding gentamicin, one pharmacokinetic study evaluated its use in burn patients, and the administered dose was 5–7 mg/kg intravenously once daily [33]. Moreover, numerous studies have evaluated a tobramycin dose of 10 mg/kg IV once daily in burn patients, and target peak concentrations have been achieved with this dose [34,66]. In the previous studies, aminoglycosides were given mainly as a combination therapy to treat *Pseudomonas aeruginosa* infections. While using aminoglycosides in burn patients, serum therapeutic drug monitoring is highly recommended [32].

### 5.2. Antifungals

#### 5.2.1. Liposomal Amphotericin B

Liposomal amphotericin B is a lipid formulation of amphotericin B with broad-spectrum antifungal activity. In burn patients, the recommended dose is 5–6 mg/kg IV once daily [51]. A case report also described successful treatment of a rare fungal infection in a burn patient using 6 mg/kg IV once daily [67].

#### 5.2.2. Azoles (Fluconazole, Voriconazole, Posaconazole, Isavuconazole)

Among azoles, **Fluconazole**: The most extensively studied antifungal in burn patients. For Candida albicans, 400 mg IV once daily is recommended, while for Candida glabrata, a higher dose of 800 mg IV once daily is preferred [35]. Evidence suggests that a standard dose of 400 mg may still achieve effective concentrations in burn patients [67]. Pharmacokinetic studies in this population demonstrate a decreased half-life, increased clearance, and increased volume of distribution for fluconazole [68].

**Voriconazole**: therapeutic drug monitoring (TDM)- guided therapy is required. Partcularly in those with altered pharmacokinetics such as burn patients.

Recommended regimen is 6 mg/kg IV every 12 h for the first two doses, followed by 4 mg/kg IV every 12 h [69].

**Posaconazole**: although it is highly used for prophylaxis in high-risk patients, it can be used also as salvage therapy for resistant fungal infection (e.g., aspergillosis). TDM-guided therapy is recommended and caution is necessary regarding drug-drug interactions.

For IV or delayed-release tablets, 300 mg every 12 h as a loading dose, then 300 mg once daily. For oral suspension, 200 mg every 6 h is suggested, with therapeutic drug monitoring [51,69].

**Isavuconazole**: Standard dosing is 200 mg IV every 8 h for six doses as a loading regimen, followed by 200 mg IV once daily. Successful use of this regimen in a burn patient with mucormycosis has been reported [6,70].

#### 5.2.3. Echinocandins

Standard dosing of anidulafungin and caspofungin can be used in burn patients. However, micafungin requires adjustment, with 150 mg IV once daily recommended [8].

## 6. Expert Opinion and Future Directions

### 6.1. Optimizing Antimicrobial Dosing and Therapeutic Drug Monitoring in Burn Patients

Improving infection management in burn patients requires focused research on how burn injuries alter the PK/PD profiles of commonly used antimicrobials. Burn-related physiological changes—such as increased capillary permeability, fluid shifts, hypermetabolism, altered protein binding, and augmented renal clearance—can significantly impact drug absorption, distribution, metabolism, and elimination. These alterations often lead to subtherapeutic antimicrobial levels if standard dosing is applied without adjustment [5].

Therefore, dosing strategies should be tailored to the unique PK/PD changes in burn patients, particularly for widely used antibiotics, as inappropriate dosing increases the risk of treatment failure and antimicrobial resistance.

Importantly, the optimal dosing and infusion regimens of newer agents, such as β-lactam/β-lactamase inhibitor combinations (e.g., ceftolozane–tazobactam, imipenem–cilastatin–relebactam, meropenem–vaborbactam), remain poorly defined in critically ill burn populations.

Future research should also prioritize the role of therapeutic drug monitoring (TDM), especially for time-dependent antibiotics where efficacy depends on maintaining free drug concentrations above the minimum inhibitory concentration (fT > MIC). Establishing standardized TDM protocols could help ensure consistent and effective antimicrobial therapy in this high-risk group.

### 6.2. Renal Function, Drug Interactions, and Burn Edema Considerations

Monitoring renal function in burn patients receiving antimicrobials is essential, particularly for agents predominantly eliminated by the kidneys. Evidence on the safety of high-dose antimicrobial use in this population remains limited; to date, only one study has specifically examined imipenem/cilastatin dose adjustment in renally impaired burn patients to reduce the risk of neurotoxicity [60]. Other nephrotoxic antimicrobials, such as vancomycin and aminoglycosides, require TDM to mitigate nephrotoxicity risk [32,63], while colistin therapy necessitates more frequent renal function monitoring [31]. In practice, renal dose adjustment recommendations for burn patients should follow the same principles as for non-burn patients.

In addition to renal considerations, potential drug–drug interactions (DDIs) must be carefully assessed when initiating antimicrobial therapy in burn patients. Azole antifungals, including voriconazole and posaconazole, are particularly prone to clinically significant interactions that may compromise efficacy or increase toxicity, highlighting the importance of DDI screening before initiation [69].

Another critical factor is the effect of burn-related edema on antimicrobial pharmacokinetics. Edematous tissue may act as a reservoir, altering drug distribution and elimination. For example, one study demonstrated prolonged ceftazidime half-life despite increased clearance, attributed to drug diffusion into edema fluid, which delayed redistribution into systemic circulation [15].

### 6.3. Antibiotic Prophylaxis in Burn Patients

Antibiotic prophylaxis is an important consideration in critically ill burn patients at high risk of infection. However, its use is indicated only in specific high-risk scenarios, and current evidence is limited, with few clinical studies providing guidance on optimal patient care [36]. This lack of robust data contributes to substantial variability in clinical practice and may lead to overuse of broad-spectrum antibiotics, increasing the risk of antimicrobial resistance. Future clinical research should focus on identifying burn patient populations that would benefit most from targeted prophylaxis.

### 6.4. Infection Prevention Strategies and Antimicrobial Stewardship

Beyond dosing and prophylaxis, infection prevention remains the cornerstone of managing burn patients. Effective strategies include meticulous wound care, strict hand hygiene, and thorough environmental decontamination [1,71]. Successful implementation requires coordinated collaboration among all healthcare professionals, including burn surgeons, physicians, pharmacists, nurses, microbiologists, and the infection control team.

In parallel, antimicrobial stewardship programs are critical for optimizing infection management in burn patients. Such programs should integrate local antibiograms, institutional formulary policies, and the specific pharmacokinetic alterations observed in burn patients to guide appropriate antimicrobial selection and dosing, thereby minimizing resistance and enhancing treatment outcomes.

## 7. Limitations

This narrative review has several limitations. First, it does not follow a systematic methodology, which may introduce selection bias in the included literature. Second, the studies reviewed are highly heterogeneous in terms of design, sample size, and methodological quality. This variability limits the ability to draw firm or generalizable conclusions. Additionally, many of the included data come from small-scale studies or individual case reports, which further reduces the strength of the evidence and its applicability to broader patient populations. As such, the findings presented should not be interpreted as prescriptive dosing recommendations, but rather as a summary of the available data to inform future research and clinical consideration. Third, this review focused on antimicrobial considerations; however, other important aspects of infection management, such as wound care techniques and nutritional status, may not be adequately addressed. Finally, multicenter clinical studies are necessary to develop comprehensive and practical clinical decision-support tools and dosing algorithms that take into account the significant pharmacokinetic changes in critically ill burn patients. Such tools will assist clinicians to explore the available evidence in decisions at the patient’s bedside, which are based on real-time clinical data, therapeutic drug monitoring results, and susceptibility patterns. Additionally, personalized antimicrobial therapy, in cooperation with antimicrobial stewardship, should be provided, especially in burn patients who exhibit significant variability in patient characteristics. This represents a promising pathway that can be achieved through precision dosing and antimicrobial stewardship to improve outcomes and preserve antibiotic efficacy in this vulnerable population.

## 8. Conclusions

In conclusion, prophylactic systemic antibiotics are generally not beneficial for burn patients, although select high-risk groups may gain limited benefit; infection control remains the cornerstone of prevention. Treatment should be guided by local resistance patterns, most commonly targeting *Pseudomonas aeruginosa* and MRSA, while antifungals are reserved for high-mortality fungal infections. Given the PK/PD alterations in burn patients, individualized antimicrobial dosing, therapeutic drug monitoring, and stewardship are crucial for optimizing outcomes. The variability in the existing evidence highlights the need for further high-quality research to strengthen the foundations of infection management in burn patients.

## Figures and Tables

**Table 1:** Summary of studies on antibiotics in burn patients.

Authors/Year	Study Design	Population and Intervention	Outcomes	Results	Levels of Evidence *	Comments
Takashi Tagami (2016) [9]	Retrospective observational study	2893 severe burn patients (burn index ≥ 10). Antibiotics initiated after day 2 or later.	28-day in-hospital mortality.	Reduction in mortality in mechanically ventilated patients. No effect in non-ventilated patients.	III	Database lacked infection data. Limited to severe burns.
Kimura A (1998) [10]	Prospective, randomized, placebo-controlled	40 Severe burns (>20% TBSA) Trimethoprim-sulfamethoxazole (n = 21) vs. placebo (n = 19) for MRSA prevention.	Incidence of MRSA pneumonia. Safety profile.	MRSA incidence reduced in TMP-SMX group (4.8% vs. 36.8%, p = 0.017). No safety differences.	II	Small sample size.
Sampol E (2000) [11]	Pharmacokinetics study	6 severe burn patients in ICU. Evaluated PK parameters over time.	PK changes post-administration.	No significant change in PK parameters [half-life (h), clearance (mL/min), area under the curve (AUC) (mg·h/L), and volume of distribution (L/kg)] between day 1 and day 3.	II	Small sample size; nature of study.
Conil JM (2007) [12]	Pharmacokinetics study	Burn patients receiving ceftazidime (CF) (n = 17): 1 g × 6 daily doses or cefepime (CE) (n = 13): 2 g × 3 daily doses) Dose variation studied.	PK variability and serum concentrations.	Significant PK differences influenced by age, ventilation, and renal clearance.	II	High variability; therapeutic monitoring recommended.
Aoki Y (2011) [13]	Case report	Single severe burn patient (≈45.5% TBSA); treated with cefepime for Pseudomonas bacteremia.	PK consultation for cefepime dosing.	Pseudomounas eradicated despite suboptimal PK profile.	IV	Case-specific; improving renal function influenced the outcome.
Alshaer M (2023) [14]	Retrospective study	19 thermal injury patients treated with beta-lactams for ≥48 h.	Therapeutic target attainment Mortality rates. Clinical cure Microbial eradication, Development of new resistance.	Therapeutic concentrations in 77%; microbial eradication in 82%. Other outcomes were similar between the groups	III	Small sample size; measured total, not free, drug levels.
Eric Dailly (2003) [15]	Pharmacokinetics study	41 burn patients. PK analysis of ceftazidime.	PK variability in non-acute burn cases.	Altered PK parameters with higher distribution and clearance.	II	A larger sample size is needed for better generalizability
Jean Marie Conil (2007) [16]	Pharmacokinetics study	50 burn patients receiving ceftazidime.	Clinical/biological factors affecting PK.	PK variability by gender, ventilation, and creatinine clearance.	II	Highlights the need for individualized dosing.
J M Conil (2007) [17]	Randomized Controlled Trial	30 serious burn patients; Ceftazidime monitoring.	Variations in serum antibiotic concentrations.	PK monitoring is required for optimized dosing.	II	High interindividual variability observed.
Bourget P (1996) [18]	Open-label PK study	10 major burn patients (≥20% TBSA) Piperacillin-tazobactam PK.	Tissue distribution Safety.	Increased clearance and volume of distribution vs. healthy subjects. No safety-related issues	II	Safe regimen; requires dose adjustment.
A. Fournier (2018) [19]	Prospective RCT	Burn ICU patients receiving antibiotics with therapeutic drug monitoring (TDM).	Impact of TDM on dosing accuracy.	TDM improved target attainment significantly.	II	Single center; small sample size.
Anna Silva Machado (2017) [20]	Retrospective study	Burn patients with healthcare-associated infections.	PK/PD monitoring vs. conventional treatment in terms of mortality and clinical cure	No significant differences in mortality; slight improvement in outcomes.	III	Retrospective design limits causality.
Doh K (2010) [21]	Pharmacokinetics study	59 burn patients on meropenem.	PK model development; dosage appropriateness.	Higher clearance and distribution than non-burn patients.	II	Tailored dosing is required for efficacy.
Messiano CG (2022) [22]	Prospective observational study	46 septic burn patients on meropenem.	Therapeutic target attainment with extended infusion.	Early-phase therapy achieved targets against intermediate MIC pathogens.	III	Variability limits universal conclusions.
Boucher BA (1990) [23]	Prospective open-label study	11 burn patients Imipenem PK evaluation.	PK variability in relation to creatinine clearance.	Clearance is highly dependent on renal function.	II	Small sample size. Single-center variability in burn severity is unreported.
Friedrich LV (1991) [24]	Prospective open-label study	8 burn patients Aztreonam PK evaluation.	Dose optimization for aztreonam.	Higher doses are needed for therapeutic concentrations.	II	Limited by small size and single-center data.
Falcone M (2021) [25]	Prospective study	8 complex burn patients treated with ceftazidime-avibactam.	PK/PD target attainment Other clinical outcomes.	Higher doses improved PK/PD targets and outcomes.	III	Small sample size. Nature of the study.
Garrelts JC (1996) [26]	Prospective multicenter trial	8 burn patients Ciprofloxacin PK evaluation.	PK comparison to healthy subjects.	Burn patients exhibited an increased clearance, volume of distribution, and shorter half-life.	II	Small sample size.
Kiser, TH (2006) [27]	Prospective, open-label, nonrandomized study	11 severe burn patients Levofloxacin PK/PD analysis.	PK variability and dosage adjustment.	Standard dosing is insufficient for PD targets.	II	Limited generalizability.
Mohr JF (2008) [28]	Prospective, open-label, pharmacokinetic study	9 thermal burn patients; single daptomycin dose.	PK/PD evaluation.	Clearance and distribution are higher vs. healthy subjects.	II	Single-dose evaluation limits clinical applicability.
Marco Falcone (2013) [29]	Retrospective study	50 MRSA bacteremia patients treated with daptomycin. 6 mg/kg (n = 31 patients) 8–10 mg/kg (n = 27 patients)	PK/PD and clinical outcomes by dose.	Higher doses improved PK/PD and cure rates.	III	Retrospective design restricts conclusions.
A.M. Lovering (2009) [30]	Open-label, multicenter study	6 major burn patients Linezolid PK evaluation.	PK variability in burn patients.	Shorter half-life and higher clearance observed.	II	Generalizability is limited by a small sample size.
Lee J, (2013) [31]	Population PK study	27 burn patients treated with colistin.	PK parameter estimation.	Burn size and renal clearance influenced dosing.	II	Significant variability; larger studies needed.
Hoey LL (1997) [32]	Prospective Observational study	33 burn patients on aminoglycosides (gentamicin or tobramycin).	Evaluation of the pharmacokinetics of single, daily-dose aminoglycosides (gentamicin or tobramycin) in burn patients.	Wide interpatient variability observed.	III	High variability affects dosing strategies.
Conil JM (2006) [33]	Prospective Clinical trial.	23 burn patients receiving amikacin.	PK variability; once-daily dosing evaluation.	Higher doses are required for efficacy.	II	Significant predictors include burn extent and time.
Vella D (2014) [34]	Retrospective study	58 burn patients on tobramycin.	PK variability Once-daily dosing feasibility.	Standard dosing is often inadequate; higher doses are needed.	III	Requires individualized monitoring.

* Quality assessment of clinical studies was based on the Oxford Centre for Evidence-Based Medicine (OCEBM) levels of evidence, which categorize studies to based on their methodological rigor and the strength of their findings. Levels of evidence by the Oxford Centre for Evidence-Based Medicine (OCEBM) II: Individual RCTs or systematic reviews of cohort studies. III: Cohort studies, case–control studies, or systematic reviews of these studies. IV: Case series, case reports, or expert opinion.

**Table 2:** Common pathogens causing burn wound infection and suggested treatment.

Organism Type	Species	Antimicrobial Treatment
Gram-positive Bacteria	MRSA	Vancomycin
	VRE	Linezolid, Daptomycin, Tigecycline
	Streptococcus	Penicillins
	Penicillin-susceptible enterococci	Penicillins
Gram-negative Bacteria	*Psuedomonas aeruginosa*	Pipercillin-tazobactam, Carbapenems, cefepime, ceftazidime
	*Acinetobacter baumanni*	Carbapenems
	(Extended-spectrum beta-lactamases) ESBL	Carbapenems
	MDR *P. aeruginosa*	Ceftolozane/Tazobactam, colistin, Ceftazidime-avibactam
Fungi	Candida	Echinocandins
		Fluconazole
		Liposomal amphotericin B
	Aspergillus	Voriconazole
		Liposomal amphotericin B
		Isavuconazole
		Posaconazole

## Data Availability

Not Applicable.

## Abbreviation

PK	Pharmacokinetics
PD	Pharmacodynamics
MRSA	Methicillin-resistant *Staphylococcus aureus*
MDR	Multidrug-resistant
Cmax	Maximum serum concentration
t1/2	Half-life
RCTs	Randomized controlled trials
TBSA	Total body surface area
TDM	Therapeutic drug monitoring
MIC	Minimum inhibitory concentration
PTA	Probability of target attainment
ARC	Augmented renal clearance
CRRT	Continuous renal replacement therapy
AUC	Area under the curve
IV	Intravenous
CK	Creatine kinase
T > MIC	Time above the minimum inhibitory concentration
CBA	Colistin Base Activity
fT > MIC	Time that free drug concentrations remain above the minimum inhibitory concentration

## References

[1] Church D., Elsayed S., Reid O., Winston B., Lindsay R. (2006). Burn wound infections. Clin. Microbiol. Rev..

[2] Ladhani H.A., Yowler C.J., Claridge J.A. (2021). Burn Wound Colonization, Infection, and Sepsis. Surg. Infect..

[3] Norbury W., Herndon D.N., Tanksley J., Jeschke M.G., Finnerty C.C. (2016). Infection in Burns. Surg. Infect..

[4] Nițescu B., Pițigoi D., Tălăpan D., Nițescu M., Aramă S.Ș., Pavel B., Streinu-Cercel A., Rafila A., Aramă V. (2023). Etiology and Multi-Drug Resistant Profile of Bacterial Infections in Severe Burn Patients, Romania 2018–2022. Medicina.

[5] Lund T., Wiig H., Reed R.K. (1988). Acute postburn edema: Role of strongly negative interstitial fluid pressure. Am. J. Physiol..

[6] Blanchet B., Jullien V., Vinsonneau C., Tod M. (2008). Influence of burns on pharmacokinetics and pharmacodynamics of drugs used in the care of burn patients. Clin. Pharmacokinet..

[7] Jeschke M.G. (2016). Postburn Hypermetabolism: Past, Present, and Future. J. Burn Care Res..

[8] Cota J.M., FakhriRavari A., Rowan M.P., Chung K.K., Murray C.K., Akers K.S. (2016). Intravenous Antibiotic and Antifungal Agent Pharmacokinetic-Pharmacodynamic Dosing in Adults with Severe Burn Injury. Clin. Ther..

[9] Tagami T., Matsui H., Fushimi K., Yasunaga H. (2016). Prophylactic Antibiotics May Improve Outcome in Patients With Severe Burns Requiring Mechanical Ventilation: Propensity Score Analysis of a Japanese Nationwide Database. Clin. Infect. Dis..

[10] Kimura A., Mochizuki T., Nishizawa K., Mashiko K., Yamamoto Y., Otsuka T. (1998). Trimethoprim-sulfamethoxazole for the prevention of methicillin-resistant *Staphylococcus aureus* pneumonia in severely burned patients. J. Trauma.

[11] Sampol E., Jacquet A., Viggiano M., Bernini V., Manelli J.C., Lacarelle B., Durand A. (2000). Plasma, urine and skin pharmacokinetics of cefepime in burns patients. J. Antimicrob. Chemother..

[12] Conil J.M., Georges B., Lavit M., Seguin T., Tack I., Samii K., Chabanon G., Houin G., Saivin S. (2007). Pharmacokinetics of ceftazidime and cefepime in burn patients: The importance of age and creatinine clearance. Int. J. Clin. Pharmacol. Ther..

[13] Aoki Y., Urakami T., Magarifuchi H., Nagasawa Z., Nagata M., Fukuoka M. (2011). The importance of pharmacokinetic consultation of cefepime treatment for *Pseudomonas aeruginosa* bacteremia: A case report of severe thermal burn injury. J. Infect. Chemother..

[14] Alshaer M., Mazirka P., Burch G., Peloquin C., Drabick Z., Carson J. (2023). Experience with Implementing a Beta-lactam Therapeutic Drug Monitoring Service in a Burn Intensive Care Unit: A Retrospective Chart Review. J. Burn Care Res..

[15] Dailly E., Pannier M., Jolliet P., Bourin M. (2003). Population pharmacokinetics of ceftazidime in burn patients. Br. J. Clin. Pharmacol..

[16] Conil J.M., Georges B., Lavit M., Laguerre J., Samii K., Houin G., Saivin S. (2007). A population pharmacokinetic approach to ceftazidime use in burn patients: Influence of glomerular filtration, gender and mechanical ventilation. Br. J. Clin. Pharmacol..

[17] Conil J.M., Georges B., Fourcade O., Seguin T., Houin G., Saivin S. (2007). Intermittent administration of ceftazidime to burns patients: Influence of glomerular filtration. Int. J. Clin. Pharmacol. Ther..

[18] Bourget P., Lesne-Hulin A., Le Reveillé R., Le Bever H., Carsin H. (1996). Clinical pharmacokinetics of piperacillin-tazobactam combination in patients with major burns and signs of infection. Antimicrob. Agents Chemother..

[19] Fournier A., Eggimann P., Pantet O., Pagani J.L., Dupuis-Lozeron E., Pannatier A., Sadeghipour F., Voirol P., Que Y.A. (2018). Impact of Real-Time Therapeutic Drug Monitoring on the Prescription of Antibiotics in Burn Patients Requiring Admission to the Intensive Care Unit. Antimicrob. Agents Chemother..

[20] Machado A.S., Oliveira M.S., Sanches C., Silva Junior C.V.D., Gomez D.S., Gemperli R., Santos S.R.C.J., Levin A.S. (2017). Clinical Outcome and Antimicrobial Therapeutic Drug Monitoring for the Treatment of Infections in Acute Burn Patients. Clin. Ther..

[21] Doh K., Woo H., Hur J., Yim H., Kim J., Chae H., Han S., Yim D.S. (2010). Population pharmacokinetics of meropenem in burn patients. J. Antimicrob. Chemother..

[22] Messiano C.G., Morales Junior R., Pereira G.O., Silva Junior E.M.D., Gomez D.S., Santos S.R.C.J. (2022). Therapeutic Target Attainment of 3-Hour Extended Infusion of Meropenem in Patients With Septic Burns. Clin. Ther..

[23] Boucher B.A., Hickerson W.L., Kuhl D.A., Bombassaro A.M., Jaresko G.S. (1990). Imipenem pharmacokinetics in patients with burns. Clin. Pharmacol. Ther..

[24] Friedrich L.V., White R.L., Kays M.B., Brundage D.M., Yarbrough D. (1991). Aztreonam pharmacokinetics in burn patients. Antimicrob. Agents Chemother..

[25] Falcone M., Menichetti F., Cattaneo D., Tiseo G., Baldelli S., Galfo V., Leonildi A., Tagliaferri E., Di Paolo A., Pai M.P. (2021). Pragmatic options for dose optimization of ceftazidime/avibactam with aztreonam in complex patients. J. Antimicrob. Chemother..

[26] Garrelts J.C., Jost G., Kowalsky S.F., Krol G.J., Lettieri J.T. (1996). Ciprofloxacin pharmacokinetics in burn patients. Antimicrob. Agents Chemother..

[27] Kiser T.H., Hoody D.W., Obritsch M.D., Wegzyn C.O., Bauling P.C., Fish D.N. (2006). Levofloxacin pharmacokinetics and pharmacodynamics in patients with severe burn injury. Antimicrob. Agents Chemother..

[28] Mohr J.F., Ostrosky-Zeichner L., Wainright D.J., Parks D.H., Hollenbeck T.C., Ericsson C.D. (2008). Pharmacokinetic evaluation of single-dose intravenous daptomycin in patients with thermal burn injury. Antimicrob. Agents Chemother..

[29] Falcone M., Russo A., Venditti M., Novelli A., Pai M.P. (2013). Considerations for higher doses of daptomycin in critically ill patients with methicillin-resistant *Staphylococcus aureus* bacteremia. Clin. Infect. Dis..

[30] Lovering A.M., Le Floch R., Hovsepian L., Stephanazzi J., Bret P., Birraux G., Vinsonneau C. (2009). Pharmacokinetic evaluation of linezolid in patients with major thermal injuries. J. Antimicrob. Chemother..

[31] Lee J., Han S., Jeon S., Hong T., Song W., Woo H., Yim D.S. (2013). Population pharmacokinetic analysis of colistin in burn patients. Antimicrob. Agents Chemother..

[32] Hoey L.L., Tschida S.J., Rotschafer J.C., Guay D.R., Vance-Bryan K. (1997). Wide variation in single, daily-dose aminoglycoside pharmacokinetics in patients with burn injuries. J. Burn Care Rehabil..

[33] Conil J.M., Georges B., Breden A., Segonds C., Lavit M., Seguin T., Coley N., Samii K., Chabanon G., Houin G. (2006). Increased amikacin dosage requirements in burn patients receiving a once-daily regimen. Int. J. Antimicrob. Agents.

[34] Vella D., Walker S.A., Walker S.E., Daneman N., Simor A. (2014). Determination of tobramycin pharmacokinetics in burn patients to evaluate the potential utility of once-daily dosing in this population. J. Burn Care Res..

[35] Barajas-Nava L.A., López-Alcalde J., Roqué i Figuls M., Solà I., Bonfill Cosp X. (2013). Antibiotic prophylaxis for preventing burn wound infection. Cochrane Database Syst. Rev..

[36] Ramos G., Cornistein W., Cerino G.T., Nacif G. (2017). Systemic antimicrobial prophylaxis in burn patients: Systematic review. J. Hosp. Infect..

[37] ISBI Practice Guidelines Committee, Steering Subcommittee, Advisory Subcommittee (2016). ISBI Practice Guidelines for Burn Care. Burns.

[38] Hill D.M., Sinclair S.E., Hickerson W.L. (2017). Rational Selection and Use of Antimicrobials in Patients with Burn Injuries. Clin. Plast. Surg..

[39] Wang Y., Beekman J., Hew J., Jackson S., Issler-Fisher A.C., Parungao R., Lajevardi S.S., Li Z., Maitz P.K.M. (2018). Burn injury: Challenges and advances in burn wound healing, infection, pain and scarring. Adv. Drug Deliv. Rev..

[40] Mir M.A., Khurram M.F., Khan A.H. (2017). What should be the antibiotic prescription protocol for burn patients admitted in the department of burns, plastic and reconstructive surgery. Int. Wound J..

[41] Moinuddin K., Alanazi D.S., Alsomali B.A., Alotaibi M., Parameaswari P.J., Ali S. (2021). Prescription Pattern of Empirical Antibiotic Therapy in the Burn Unit of a Tertiary Care Setting in the Kingdom of Saudi Arabia. J. Pharm. Bioallied Sci..

[42] Khan T.M., Kok Y.L., Bukhsh A., Lee L.H., Chan K.G., Goh B.H. (2018). Incidence of methicillin resistant *Staphylococcus aureus* (MRSA) in burn intensive care unit: A systematic review. Germs.

[43] Saaiq M., Zaib S., Ahmad S. (2012). Early excision and grafting versus delayed excision and grafting of deep thermal burns up to 40% total body surface area: A comparison of outcome. Ann. Burns Fire Disasters.

[44] Roberts J.A., Abdul-Aziz M.H., Lipman J., Mouton J.W., Vinks A.A., Felton T.W., Hope W.W., Farkas A., Neely M.N., Schentag J.J. (2014). Individualised antibiotic dosing for patients who are critically ill: Challenges and potential solutions. Lancet Infect. Dis..

[45] Cambiaso-Daniel J., Gallagher J.J., Norbury W., Finnerty C.C., Herndon D., Culnan D.M., Herndon D.N. (2018). Treatment of Infection in Burn Patients. Total Burn Care.

[46] Frederick A.B., Skidmore S.H., Lesher A.P., Kahn S.A., Mittal R. (2025). Invasive Fungal Infection Increases Mortality Risk After Burn Injury. J. Burn Care Res..

[47] Gur I., Zilbert A., Toledano K., Roimi M., Stern A. (2024). Clinical impact of fungal colonization of burn wounds in patients hospitalized in the intensive care unit: A retrospective cohort study. Trauma Surg. Acute Care Open.

[48] Nitsani Y., Michael T., Halpern D., Hasidim A.A., Sher M., Givoli Vilensky R., Krieger Y., Silberstein E., Shoham Y. (2023). Blood Stream Infections in Burns: A 14-Year Cohort Analysis. Life.

[49] Pappas P.G., Kauffman C.A., Andes D.R., Clancy C.J., Marr K.A., Ostrosky-Zeichner L., Reboli A.C., Schuster M.G., Vazquez J.A., Walsh T.J. (2016). Clinical Practice Guideline for the Management of Candidiasis: 2016 Update by the Infectious Diseases Society of America. Clin. Infect. Dis..

[50] Patterson T.F., Thompson G.R., Denning D.W., Fishman J.A., Hadley S., Herbrecht R., Kontoyiannis D.P., Marr K.A., Morrison V.A., Nguyen M.H. (2016). Practice Guidelines for the Diagnosis and Management of Aspergillosis: 2016 Update by the Infectious Diseases Society of America. Clin. Infect. Dis..

[51] Walraven C., Mercier R.-C., Lee S. (2011). Antifungal Pharmacokinetics and Dosing Considerations in Burn Patients. Curr. Fungal Infect. Rep..

[52] Bonapace C.R., White R.L., Friedrich L.V., Norcross E.D., Bosso J.A. (1999). Pharmacokinetics of cefepime in patients with thermal burn injury. Antimicrob. Agents Chemother..

[53] Le Floch R., Arnould J.F., Pilorget A., Dally E., Naux E. (2010). Antimicrobial blood concentrations in burns. A five years’ retrospective survey. Pathol. Biol..

[54] Walstad R.A., Aanderud L., Thurmann-Nielsen E. (1988). Pharmacokinetics and tissue concentrations of ceftazidime in burn patients. Eur. J. Clin. Pharmacol..

[55] Jeon S., Han S., Lee J., Hong T., Paek J., Woo H., Yim D.S. (2014). Population pharmacokinetic analysis of piperacillin in burn patients. Antimicrob. Agents Chemother..

[56] Olbrisch K., Kisch T., Thern J., Kramme E., Rupp J., Graf T., Wicha S.G., Mailänder P., Raasch W. (2019). After standard dosage of piperacillin plasma concentrations of drug are subtherapeutic in burn patients. Naunyn Schmiedebergs Arch. Pharmacol..

[57] Ramon-Lopez A., Allen J.M., Thomson A.H., Dheansa B.S., James S.E., Hanlon G.W., Stewart B., Davies J.G. (2015). Dosing regimen of meropenem for adults with severe burns: A population pharmacokinetic study with Monte Carlo simulations. J. Antimicrob. Chemother..

[58] Selig D.J., Akers K.S., Chung K.K., Pruskowski K.A., Livezey J.R., Por E.D. (2022). Meropenem pharmacokinetics in critically ill patients with or without burn treated with or without continuous veno-venous haemofiltration. Br. J. Clin. Pharmacol..

[59] Corcione S., D’Avolio A., Loia R.C., Pensa A., Segala F.V., De Nicolò A., Fatiguso G., Romeo M., Di Perri G., Stella M. (2020). Pharmacokinetics of meropenem in burn patients with infections caused by Gram-negative bacteria: Are we getting close to the right treatment?. J. Glob. Antimicrob. Resist..

[60] Gomez D.S., Sanches-Giraud C., Silva C.V., Oliveira A.M., da Silva J.M., Gemperli R., Santos S.R. (2015). Imipenem in burn patients: Pharmacokinetic profile and PK/PD target attainment. J. Antibiot..

[61] Li S., Xie F. (2019). Population pharmacokinetics and simulations of imipenem in critically ill patients undergoing continuous renal replacement therapy. Int. J. Antimicrob. Agents.

[62] Carter B.L., Damer K.M., Walroth T.A., Buening N.R., Foster D.R., Sood R. (2015). A Systematic Review of Vancomycin Dosing and Monitoring in Burn Patients. J. Burn Care Res..

[63] Ortwine J.K., Pogue J.M., Faris J. (2015). Pharmacokinetics and pharmacodynamics of antibacterial and antifungal agents in adult patients with thermal injury: A review of current literature. J. Burn Care Res..

[64] Huang Y., Lv G., Hu L., Wu Y., Guo N., Zhu Y., Ding L., Li Q., Liu S., Yang Y. (2020). Efficacy and Safety of High Vs Standard Daptomycin Doses Examined in Chinese Patients With Severe Burn Injuries by Pharmacokinetic Evaluation. J Burn Care Res..

[65] Mokline A., Gharsallah L., Rahmani I., Gaies E., Tabelsi S., Messadi A.A. (2018). Pharmacokinetics and pharmacodynamics of Linezolid in burn patients. Ann. Burns Fire Disasters.

[66] Lee C., Walker S.A.N., Walker S.E., Seto W., Simor A., Jeschke M. (2017). A prospective study evaluating tobramycin pharmacokinetics and optimal once daily dosing in burn patients. Burns.

[67] Han S., Kim J., Yim H., Hur J., Song W., Lee J., Jeon S., Hong T., Woo H., Yim D.S. (2013). Population pharmacokinetic analysis of fluconazole to predict therapeutic outcome in burn patients with Candida infection. Antimicrob. Agents Chemother..

[68] Santos S.R., Campos E.V., Sanches C., Gomez D.S., Ferreira M.C. (2010). Fluconazole plasma concentration measurement by liquid chromatography for drug monitoring of burn patients. Clinics.

[69] Musick K.L., Jones S.L., Norris A.M., Hochstetler L.J., Williams F.N., McKinzie B.P. (2022). Evaluation of Voriconazole and Posaconazole Dosing in Patients with Thermal Burn Injuries. J. Burn Care Res..

[70] Galvez A., Lipka O., Haith L., Scantling D., Kaplan M., Patton M., Guilday R. (2017). Treatment of Invasive Mucormycosis with Intravenous Isavuconazonium and Topical Amphotericin B in a Renal-Impaired Patient: Case Report and Review of the Literature. Surg. Infect. Case Rep..

[71] Weber J., McManus A., Nursing Committee of the International Society for Burn Injuries (2004). Infection control in burn patients. Burn. J. Int. Soc. Burn Inj..

